# Combining Transcriptome and Hormone-Targeted Metabolome Analyses to Dissect the Regulatory Mechanisms Underlying Wheat Peduncle Elongation

**DOI:** 10.3390/plants14111611

**Published:** 2025-05-25

**Authors:** Huifang Hao, Lu Chen, Zhiyang Cao, Xiujuan Jin, Feng Guo, Zerui Shi, Jinwen Yang, Juan Lu, Daizhen Sun

**Affiliations:** College of Agriculture, Shanxi Agricultural University, Jinzhong 030801, China; jyhf2009@163.com (H.H.);

**Keywords:** wheat, peduncle, *eui* mutant, transcriptomics, hormone-targeted metabolomics

## Abstract

Wheat is an important global food crop. The peduncle significantly impacts the plant’s height, architecture, and yield, and understanding its genetic mechanisms is crucial not only for improving wheat’s architecture but also for enhancing its yield. In this study, we identified an *elongated uppermost internode (eui)* mutant in the EMS-induced progeny of Jinmai 90 (JM90). We conducted phenotypic identification, genetic analysis, and cytological observation combined with transcriptome and targeted hormone metabolism analysis and compared the differences between the *eui* mutant and the wild-type (WT). The results indicated that an incompletely dominant gene mutation caused the *eui* mutant to display significant elongation of the peduncle and an increase in the plant height. This was attributed to the considerable elongation of parenchyma cells, while no significant differences were noted in other internodes. These traits were accompanied by an increase in the spikelets per spike and grains per spike. Subsequently, transcriptome and targeted hormone metabolome sequencing were performed and identified 15,969 differentially expressed genes (DEGs) and 27 hormone-related differentially accumulated metabolites (DAMs). KEGG enrichment analysis indicated that the DEGs in MS1_VS_WS1 were significantly enriched in two pathways: those related to tryptophan metabolism and diterpenoid biosynthesis. Analysis indicated that the peduncle elongation caused by the *eui* mutant is primarily regulated by auxin. This study offers a foundation for the exploration and cloning of genes associated with the peduncle, establishing a theoretical basis for understanding the molecular mechanisms behind wheat peduncle elongation and for developing ideal plant types and breeding high-yield varieties.

## 1. Introduction

Wheat (*Triticum aestivum* L.) is a vital global food crop, supplying 55% of human energy consumption [1,2,3]. The peduncle is the distance from the base of the wheat ear to the first internode, which primarily serves as a conduit for transporting photosynthetic products from the leaves to the ears and is a significant trait as it influences both the height and type of the wheat plant. Additionally, it is an important source of energy during the filling stage, which can significantly impact the wheat yield [4]. Furthermore, longer peduncles may enhance air circulation, reduce the spread of pathogens, and improve the plant’s resistance to diseases [5]. However, varieties with longer peduncles often exhibit an increased plant height [6], which can easily lead to lodging. Therefore, breeding varieties with longer peduncles but a relatively low plant height will improve the internode configuration and benefit crop production practices. The relationship between the peduncle length and plant height is complex; in some cases, the peduncle changes in tandem with the plant height [7], while in others, it appears to be independent of it [8]. The associated regulatory mechanisms remain unclear. Therefore, elucidating the genetic regulatory networks that control peduncle development is crucial for enhancing the wheat yield and optimizing the plant architecture.

The use of transcriptomics allows for the sequence and expression information of nearly all transcripts from a specific cell or tissue in a given state to be comprehensively and rapidly obtained. For instance, transcriptomes have been utilized to uncover genes associated with stem elongation [9,10,11,12]. Metabolomics involves the simultaneous qualitative and quantitative analysis of all the low-molecular-weight metabolites within an organism or cell during a specific physiological period. Integrating these analyses enables a concurrent understanding of gene regulatory mechanisms and changes in the metabolite abundance within cells, providing comprehensive insights into the systemic alterations within organisms. For instance, transcriptomics and metabolomics have been used to elucidate the stem development mechanisms of crops [13,14].

Studies have shown that plant hormones are integral to regulating the elongation of crop stems [15]. Auxin plays a crucial role in plant growth by influencing cell elongation, division, and differentiation, promoting cell elongation by facilitating cell wall loosening and stimulating the synthesis of proteins and nucleic acids [16,17]. For example, the overexpression of *OsYUCCA2* promotes rice (*Oryza sativa* L.) growth [18]. Auxin response factors *(ARFs)* also impact plant stem growth [19,20]. The *VT2* mutation in corn substantially impacts both nutritional and reproductive growth processes [21]. The *brachytic2 (Br2)* gene mutant can reduce the plant height and ear height in maize (*Zea mays* L.) and encodes an ATP-binding cassette auxin transporter [22]. Furthermore, auxin can synergize with other hormones to enhance stem elongation [23]. Auxin induces an increase in the gibberellin content in deep rice, thereby promoting stem elongation [24]. Gibberellin exerts a direct influence on plant stem elongation [25]. The loss-of-function mutation of the “Green Revolution gene” *OsGA20ox2* causes semi-dwarfism [26]. Mutations in the *GA13ox* gene result in elongated peduncles in rice [27]. Some studies have also suggested that brassinosteroids play an important role in plant stem elongation. Mutations in the BR receptor gene *OsBRI1* typically result in the second internode being significantly shorter than the first, leading to dwarfism in rice [28]. However, the regulation of wheat peduncle elongation by these hormones remains poorly understood.

In this study, we subjected the *eui* mutant, derived from the EMS mutagenesis of JM90 and characterized by an elongated uppermost internode, to phenotypic identification and cytological observations, comparing it with the WT. Moreover, we analyzed the transcriptome and hormone-targeted metabolome of peduncles at two stages of elongation: S1 (prior to full spike emergence) and S2 (ten days post-S1, when the spikes fully emerged). Our objective was to investigate the mechanisms responsible for the morphological changes in these mutants, with a focus on metabolic pathways closely associated with peduncle elongation, in order to identify key genes and metabolites. This research aimed to establish a theoretical foundation for understanding the molecular mechanisms behind wheat peduncle elongation and ensuring the ideal plant type in wheat.

## 2. Results

### 2.1. The Manifestations of the Elongated Uppermost Internode Mutant (eui)

**Figure 1:**
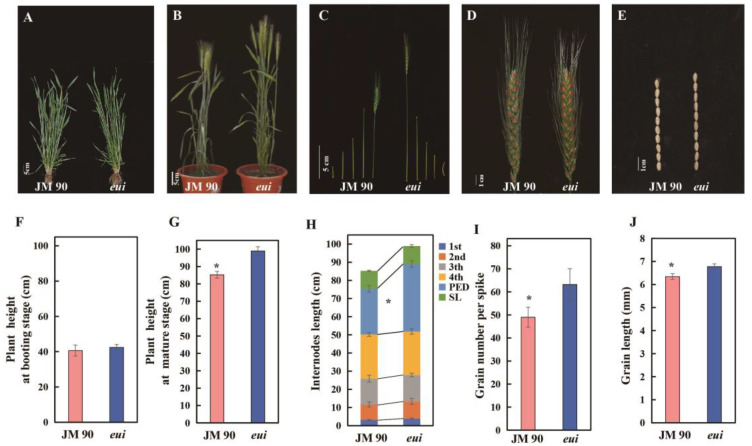
Phenotypic characteristics of the wild-type (JM90) and the mutant *(eui)*: (**A**) the plant height at the booting stage; (**B**) the plant height at the mature stage; (**C**) the internodes at the mature stage; (**D**) the spike; (**E**) the grain length; (**F**–**J**) histograms of the corresponding indices. The data represent the average from 10 plants, with an * indicating a significant difference between the wild-type JM90 and the mutant (*p* < 0.05, *t*-test).

The *eui* mutant was derived from the common wheat JM90 through EMS mutagenesis. At the booting stage, there were no significant differences in the plant height (PH) between *eui* and JM90 plants (Figure 1A,F), while at the maturity stage, the PH of *eui* plants was significantly higher than that of JM90 plants (Figure 1B,G). Further analysis revealed that both had five internodes, with the differences primarily present in the peduncle. The peduncle of *eui* plants was significantly longer than that of JM90 plants, while the lengths of the other internodes were not significantly different (Figure 1C,H). A comparison of the yield traits showed that the number of spikelets per spike in *eui* plants was greater than in JM90 plants (Figure 1D), resulting in a significant increase in the number of grains per spike (Figure 1I). The grain length was also significantly longer than that of JM90 plants (Figure 1E,J), but there were no significant differences in other agronomic traits (Supplementary Figure S1). Thus, *eui* is characterized by a significant plant height increase, primarily due to a longer peduncle, and a significant increase in the grains per spike.

### 2.2. Genetic Analysis of eui

To elucidate the genetic basis underlying the elongated peduncle phenotype, the *eui* mutant was crossed with AK58, a dwarf variety, and the peduncle length approached that of JM90. The sequence was determined, which will facilitate the localization of the mutant gene in the future. All F_1_ individuals exhibited a phenotype characterized by a tall height with a long peduncle, indicating the dominant nature of the elongated peduncle trait. Subsequently, the F_1_ individuals were self-pollinated to generate an F_2_ population. In the AK58×*eui* cross, 521 plants were similar to the wild-type, 476 plants were tall with long peduncles, and 1035 exhibited an intermediate phenotype, fitting a 1:2:1 ratio (χ2 = 2.704 < χ2 (0.05,1) = 3.84) (Table 1). These results suggested that the elongated peduncle phenotype of *eui* plants was caused by an incompletely dominant mutation at a single locus.

### 2.3. Observation of Cell Structure

**Figure 2:**
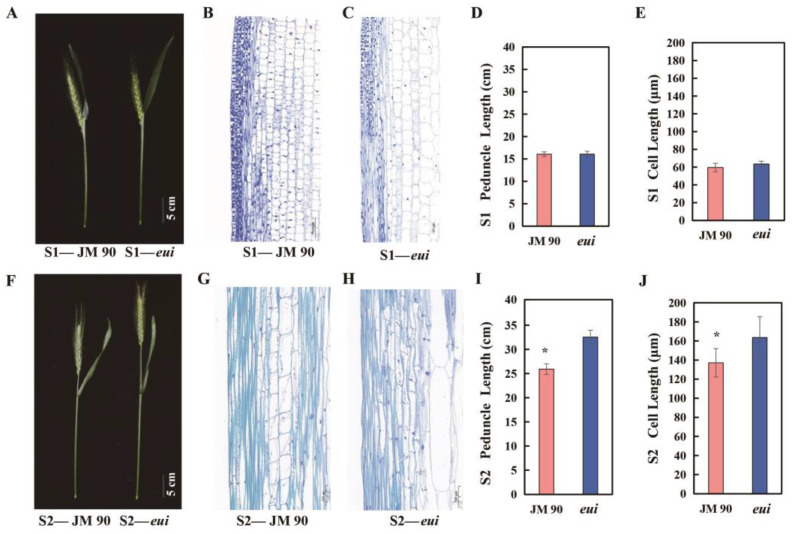
The elongation state of the peduncle and cell microstructure in the WT and *eui* plants at two stages. (**A**) Spike extension state of the WT and *eui* plants at the S1 stage. (**B**,**C**) Longitudinal section microstructures of the peduncles of WT and *eui* plants at the S1 stage, respectively. (**D**) Histogram of the peduncle length at the S1 stage. (**E**) Histogram of the cell length at the S1 stage. (**F**–**J**) S2 stage. The data are the average from 10 cells; * indicates a significant difference between the mutant and the WT (*p* < 0.05, *t*-test).

To understand the cytological basis of the significant *eui*-caused peduncle elongation, the middle parts of the peduncles from the *eui* mutant and WT plants were cut into 1 cm segments at the S1 and S2 stages to prepare paraffin sections. The sizes of the parenchyma cells were then measured. At the S1 stage, there was no significant difference in the peduncle elongation (Figure 2A,D). The cell microstructure revealed that the cells in *eui* plants were longer than those in the WT, but the difference did not reach statistical significance (Figure 2B,C,E). However, at the S2 stage, the peduncle of *eui* plants was significantly longer than that of WT plants (Figure 2F,I) and the cell length in *eui* plants was 19.08% greater than in WT plants (Figure 2G,H,J). This indicated that the growth of the *eui* peduncle was due to the elongated internode cell length, with cells elongating rapidly during the heading stage. When there was a significant difference in the plant height, the cell length also showed a significant difference.

### 2.4. Dynamic Transcriptional Variations in eui and WT Wheat Peduncles

**Figure 3:**
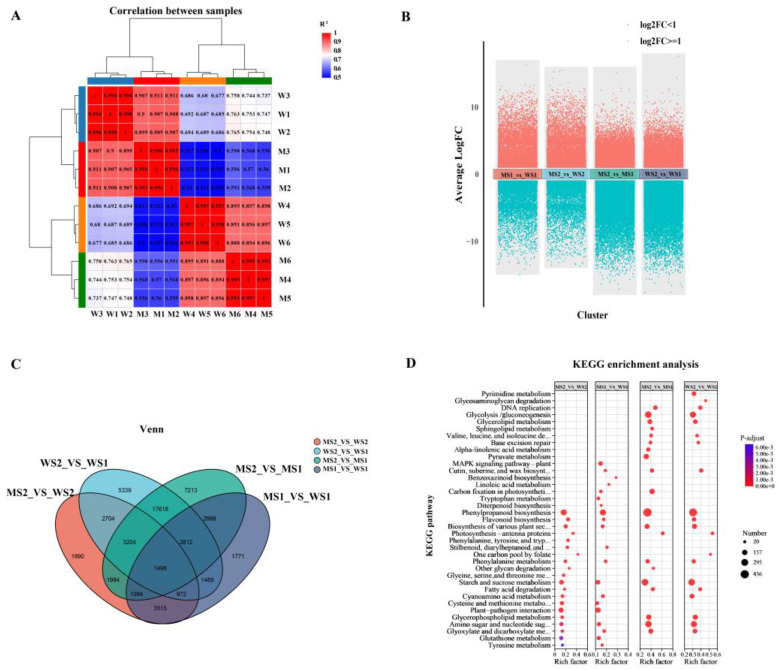
An overview of the *eui* and WT peduncle transcriptomes. (**A**) Correlation coefficient heatmaps for 12 samples: On the right and lower sides are the sample names, on the left and upper sides are the sample clusters, and the squares of different colors represent the correlation between the two samples. The correlation coefficient is shown by a color gradient ranging from low (blue) to high (red). (**B**) Volcano plot of the differentially expressed genes (DEGs), in which red represents upregulation and blue represents downregulation. (**C**) Venn diagram of the DEGs: the circles of different colors represent different gene sets, and the values represent the number of common and unique genes between different gene sets. (**D**) Top twenty pathways enriched according to the KEGG for the DEGs: The vertical axis represents the pathway name, and the horizontal axis represents the ratio of the number of genes enriched in the pathway (sample number) to the number of annotated genes (background number). The larger the rich factor, the greater the degree of enrichment. The size of the point indicates the number of genes in that pathway, and its color corresponds to different p-adjust ranges.

A total of 12 samples from two materials and two periods yielded 188.33 Gb of Clean Data, with each sample producing over 14.36 Gb. Of this, 93.67–94.56% aligned with the Chinese Spring reference genome (IWGSC_RefSeq_v2.1) (Supplementary Table S1). The correlation between the biological replicates was strong, with correlation coefficients ranging from 0.993 to 1.00. Samples within each group and between groups showed significant separation, indicating good consistency within groups and notable differences between the two varieties (Figure 3A). Six genes were randomly selected for RT-qPCR validation, and the majority of the expression trends matched the RNA-seq results (Supplementary Figure S2). These findings suggest that the test sampling was appropriate and that the RNA-seq data quality was reliable.

The comparisons MS1_vs_WS1, MS2_vs_WS2, MS2_vs_MS1, and WS2_vs_WS1 revealed 16,007 (8444 upregulated and 7563 downregulated), 16,851 (9901 upregulated and 6950 downregulated), 38,277 (15,879 upregulated and 22,398 downregulated), and 35,636 (14,259 upregulated and 21,377 downregulated) differentially expressed genes (DEGs) (Figure 3B,C). Subsequently, the Kyoto Encyclopedia of Genes and Genomes (KEGG) pathway enrichment analysis of the *eui* and WT samples indicated that the DEGs were primarily enriched in several pathways, including those related to “Phenylpropanoid biosynthesis”, “Flavonoid biosynthesis”, “Biosynthesis of various plant secondary metabolites”, and “Phenylalanine metabolism”. In the MS1_vs_WS1 comparison, the DEGs were also notably enriched in two hormone signaling pathways, namely those related to “tryptophan metabolism” and “diterpenoid biosynthesis”, which corresponded to the auxin and gibberellin synthesis pathways, respectively (Figure 3D, Supplementary Table S2).

### 2.5. Dynamic Metabolomic Changes in eui and WT Peduncles

**Figure 4:**
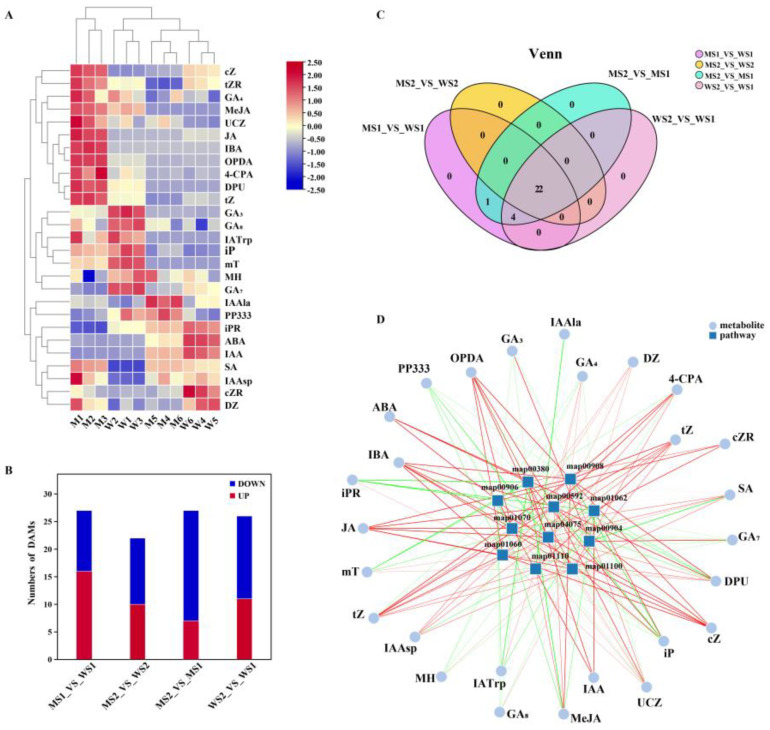
An overview of the *eui* and WT peduncle metabolomes. (**A**) Heatmap of the DAMs detected and related to hormones; on the right side are the metabolites’ names, on the lower side are the sample names, on the left and upper sides are the sample clusters, and the relative level of accumulation is shown by a color gradient ranging from low (blue) to high (red). (**B**) Column chart of the DAMs, in which red represents upregulation and blue represents downregulation. (**C**) Venn diagram of the DAMs; the circles of different colors represent different metabolite sets, and the values represent the number of common and unique metabolites between different sets. (**D**) Correlation network diagram of the DAMs and metabolic pathways; the dots represent metabolites, the squares represent metabolic pathways, and the lines represent the correlation between them. The darker the line color is, the greater the correlation is. Green represents a negative correlation, red represents a positive correlation, and the thicker the line is, the smaller the *p*-value is.

Considering the significant role of plant hormones in plant stems, the DEGs of *eui* plants and the WT were notably enriched in hormone-related pathways. A targeted hormone metabolomics analysis of the peduncles from *eui* and WT plants at the S1 and S2 stages was conducted using liquid chromatography–mass spectrometry (LC-MS) techniques. We measured 41 hormone metabolites, and a total of 27 differentially accumulated metabolites (DAMs) were observed, encompassing auxins, gibberellins, cytokinins, jasmonic acids, salicylic acid, and abscisic acid (refer to Supplementary Table S3). Cluster analysis demonstrated that the three replicates within each group clustered, and significant differences were observed between the four groups (Figure 4A). This suggests that the metabolomics data exhibited good repeatability and high reliability. The comparisons MS1_vs_WS1, MS2_vs_WS2, MS2_vs_MS1, and WS2_vs_WS1 revealed 27 (16 upregulated and 11 downregulated), 22 (10 upregulated and 12 downregulated), 27 (7 upregulated and 20 downregulated), and 26 (11 upregulated and 15 downregulated) DAMs (Figure 4B,C). KEGG enrichment analysis indicated that these DAMs were predominantly enriched in 11 metabolic pathways, including those corresponding to tryptophan metabolism (map00380), zeatin biosynthesis (map00908), carotenoid biosynthesis (map00906), alpha-linolenic acid metabolism (map00592), the biosynthesis of terpenoids and steroids (map01062), the biosynthesis of plant hormones (map01070), plant hormone signal transduction (map04075), diterpenoid biosynthesis (map00904), the biosynthesis of plant secondary metabolites (map01060), the biosynthesis of secondary metabolites (map01110), and metabolic pathways (map01100) (Figure 4D).

Considering the pivotal roles of auxin and gibberellin in plant stem elongation and the significant enrichment of DEGs in biosynthesis pathways related to these two hormones, we conducted a detailed analysis of the DEGs and DAMs associated with auxin and gibberellin.

### 2.6. Differences in Detection of Auxin Between eui and WT

**Figure 5:**
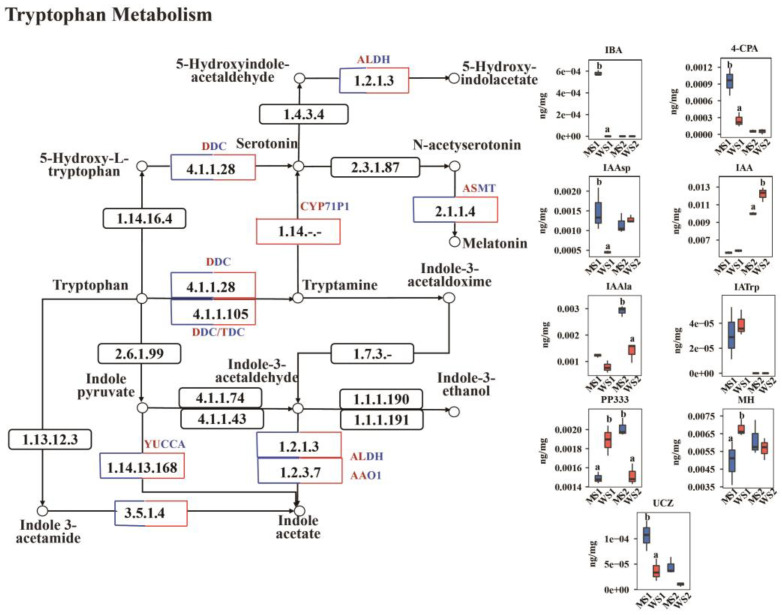
Schematic representation of the tryptophan biosynthesis pathway, which is involved in auxin synthesis. Note: Squares and circles denote genes and metabolites, respectively. Red squares indicate upregulated genes, blue squares indicate downregulated genes, and squares with both colors represent genes that were upregulated and downregulated at the S1 stage. *DDC/TDC: tryptophan decarboxylase; CYP71P1: cytochrome P450 monooxygenase*, *family 71*, *subfamily P1; ALDH: aldehyde dehydrogenase; ASMT: N-acetylserotonin O-methyltransferase; YUCCA: flavin monooxygenase; AAO1: aldehyde oxidase 1*. The inset bar plots display the abundance of the corresponding metabolites. MS1 and WS1 represent the mutant and WT at the S1 stage, respectively, while MS2 and WS2 represent the mutant and WT at the S2 stage. Statistically significant metabolites were identified using Student’s *t*-test (*p* < 0.05) and corrected for multiple comparisons. Different letters represent a significant difference between the mutant and the wild-type in the same stage. IBA: 3-indolebutyric acid; 4-CPA: 4-chlorophenoxyacetic acid; IAAsp: indole-3-acetyl-L-aspartic acid; IAA: indole-3-acetic acid; IAAla: N-(3-indolylacetyl)-L-alanine; PP333: paclobutrazol; MH: maleic hydrazide; IATrp: indole-3-acetyl-L-tryptophan; UCZ: uniconazole.

The transcriptomic results indicated an abundance of DEGs in the tryptophan metabolism pathway, which is the primary pathway for auxin biosynthesis. Some DAMs related to auxins were also identified. Thus, we analyzed the DEGs and DAMs in this pathway (Figure 5, Supplementary Table S4). Significant changes were observed in the MS1_vs_WS1 comparison, particularly for genes that were upregulated in the mutant, such as the *tryptophan decarboxylase (DDC/TDC)*, *N-acetylserotonin O-methyltransferase (ASMT)*, *CYP71P1*, and E3.5.1.4 genes. For instance, *TraesCS7D03G1269200*, *TraesCS4B03G0980700*, *TraesCS6B03G0312400*, and *TraesCS2A03G1357400* were upregulated 8.20-, 16.23-, 7.83-, and 1.89-fold, respectively, implying they have positive regulatory roles during the peduncle elongation process. By contrast, some genes were downregulated, such as the *aldehyde dehydrogenase (ALDH)* and *flavin monooxygenase (YUCCA)* genes. For example, *TraesCS6A03G0768200LC* and *TraesCS3A03G0706200* were downregulated 3.09- and 5.68-fold, respectively. In addition, some auxin-related genes related to lignin synthesis, such as *caffeic acid O-methyltransferase (OMT/COMT)*, *TraesCS2B03G0142800*, and *TraesCS5D03G1074800*, were significantly upregulated by 6.50- and 5.15-fold, respectively. Furthermore, the growth-promoting compounds IBA, 4-CPA, and IAAsp were significantly accumulated, whereas the growth-inhibiting substances PPP333 and MH were significantly reduced in MS1. In the MS2_vs_WS2 comparison, most *DDC/TDC*, *ASMT*, and *OMT/COMT* genes were upregulated, and some exhibited greater than 6-fold upregulation. The metabolomics results showed that IAAla and PP333 significantly increased in the mutants, while IAA significantly decreased in MS2. This indicates that the elongation process of the mutant peduncles consumed more auxin.

### 2.7. Differences in Detection of Gibberellin Between eui and WT

**Figure 6:**
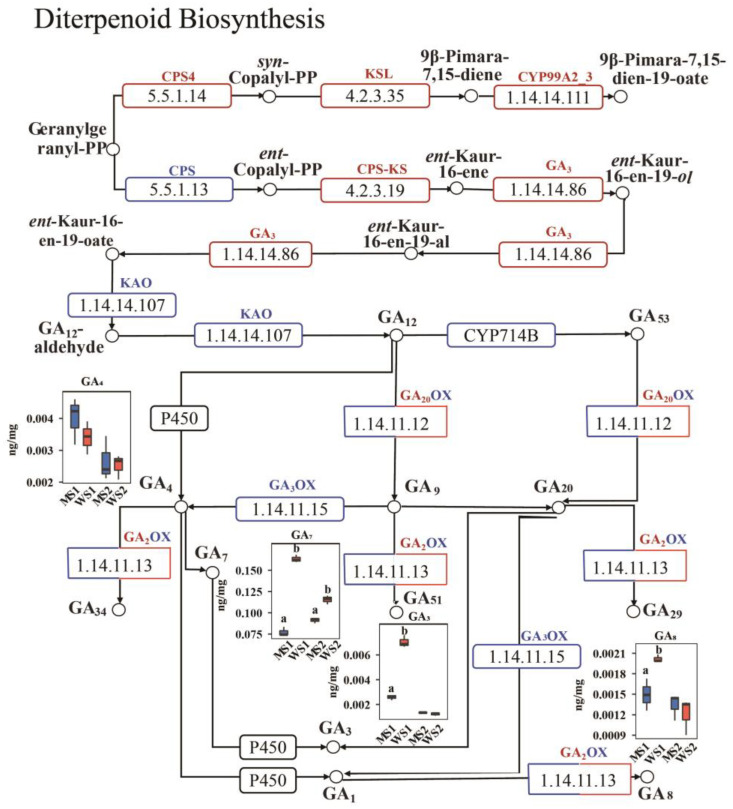
Schematic representation of the diterpenoid biosynthesis pathways involved in gibberellin synthesis. Note: Refer to Figure 5. Different letters represent a significant difference between the mutant and the wild-type in the same stage. *CPS: copalyl diphosphate synthase; KSL: ent-kaurene synthase-like; CPS-KS: ent-kaurene synthase; CYP99A: cytochrome P450 99A-like; GA_3_: ent-kaurene oxidase; KAO: ent-kaurenoic acid oxidase; CYP714B: cytochrome P450 714B-like; GA_2_OX: gibberellin 2-beta-dioxygenase; GA_3_OX: gibberellin 3-beta-dioxygenase; GA_20_OX: gibberellin 20 oxidase*; GA: gibberellin A.

The transcriptomic results indicated an abundance of DEGs in the diterpenoid biosynthesis pathway, which is upstream of the gibberellin biosynthesis pathway. Additionally, four gibberellin-derived DAMs were also identified in the metabolome. Consequently, we constructed a regulatory network that incorporated both the DEGs and DAMs within this pathway (Figure 6, Supplementary Table S5). Significant changes were noted in the MS1_vs_WS1 comparison, with certain genes demonstrating downregulation, such as *copalyl diphosphate synthase (CPS)*, *cytochrome P450 714B1-like (CYP714B)*, *ent-kaurenoic acid oxidase (KAO)*, and most *GA_20OX_* and *GA_3OX_* genes. However, more genes were upregulated, including *copalyl diphosphate synthase 4 (CPS4)*, *cytochrome P450 99A2-like (CYP99A2_3)*, *ent-kaurene synthase (CPS-KS)*, *ent-kaurene synthase-like (KSL)*, E1.14.14.86 *(GA_3_)*, and most *GA_2OX_* genes. Furthermore, the levels of GA_3_, GA_7,_ and GA_8_, known for their roles in stem elongation, were significantly downregulated, whereas GA_4,_ which promotes flowering, was slightly upregulated at the S1 stage. In the MS2_vs_WS2 comparison, the expression level of GA_20OX_ remained lower, while that of GA_2OX_ remained higher. Notably, at the S2 stage, the mutant GA_4_, GA_8_, and GA_3_ contents were higher than those of the WT; however, this difference did not reach statistical significance, and the GA_7_ content in the mutant plants remained significantly lower than that in the WT.

### 2.8. Differences in Detection of Other Hormones Between eui and WT

The DEGs and DAMs of other hormones in the *eui* mutant and WT peduncles at the two developmental stages were also compared (Supplementary Figure S3, Supplementary Table S6). Brassinosteroids (BRs) are essential for regulatory processes in plant stems. We identified several BR-related DEGs, but no corresponding metabolites were detected. Three *deetiolated 2 (DET2)* genes were upregulated in the *eui* mutant at both the S1 and S2 stages, whereas two *brassinazole-resistant 1_2 (BZR1_2)* genes and one *brassinosteroid-insensitive 1 (BRI1)* gene exhibited upregulated expression at the S1 stage. By contrast, the expression of *brassinosteroid-insensitive 2 (BIN2)* was significantly downregulated at the S2 stage. Cytokinins (CYTs) also play a role in stem elongation. In the *eui* mutant, most of the genes involved in the CYT signaling pathway, including *cis-zeatin-O-glucosyltransferase (CISZOG)*, *UDP glycosyltransferase (UGT73C)*, *arabidopsis histidine-phosphotransfer proteins (AHPs)*, and *cytokinin oxidase/dehydrogenase (CKX)*, were significantly upregulated at stage S1. Correspondingly, the metabolites tZ, tZR, cz, DZ, and DPU also showed significant accumulation, whereas the accumulation of iP, iPR, and mT was significantly reduced. Additionally, most genes in the jasmonic acid (JA) signaling pathway were significantly upregulated, resulting in significant JA, MeJA, and OPDA accumulation. *Pathogenesis-related protein (PR1)* genes associated with the SA signaling pathway were also significantly upregulated, with significant salicylic acid (SA) accumulation. By contrast, the *protein phosphatase 2C (PP2C)* and *SNF1-related protein kinase 1 (SnRK2) genes*, which are involved in the *abscisic acid (ABA)* signaling pathway, were downregulated to varying degrees at both stages, with a significant reduction in ABA accumulation observed at stage S2.

## 3. Discussion

### 3.1. eui Is a Mutant Causing Significant Peduncle Elongation and a Significant GNS Increase

The peduncle of gramineous crops plays a crucial role in providing mechanical support and supplying nutrients to the panicles. It is also a key indicator for enhancing plant varieties. In this study, we observed that the peduncle of the *eui* mutant was significantly elongated—a phenotype consistent with the known effects of the *eui* gene on the uppermost internode in rice. However, the other internodes did not show significant differences compared with the WT. Therefore, the *eui* mutant is a valuable tool for investigating the regulatory mechanisms governing the wheat peduncle.

To date, research on the wheat peduncle has primarily focused on Quantitative Trait Loci (QTLs) distributed across 21 chromosomes, most of which are associated with the pH [29,30,31], while only a few remain independent of the PH [32]. Research has indicated that the peduncle length plays a significant role in determining the yield of wheat crops. For instance, Wang proposed that photosynthesis in the exposed peduncle and flag leaf sheath contributes significantly to the grain dry mass, with estimates suggesting that its contribution accounts for about 9–12% of the total [33]. This is supported by research indicating that the photosynthetic activity in these plant parts is crucial for dry matter accumulation and the grain yield, as seen in studies on rice leaf sheaths and wheat flag leaves. Selection for an increased peduncle length in later generations may indirectly improve the yield [34]. In this study, the mutant *eui* exhibited more grains per spike (GNS), consistent with previous research. Varieties with longer peduncles not only enhance the plant architecture and boost the photosynthetic efficiency of the lower leaves but also continue to synthesize photosynthetic products as a “source” after the flag leaf begins to senesce during the filling stage, ultimately leading to an increased yield. Notably, an excessively long peduncle can also negatively impact the yield, as longer peduncles are often accompanied by a greater plant height [35], which may lead to lodging. Therefore, breeding varieties with longer peduncles is only beneficial for production within a certain plant height range.

The mutant identified in this study exhibited significant peduncle elongation without notable changes in other internodes. We also utilized this mutant to conduct a population-based preliminary genetic analysis of the mutant gene and found that this mutant trait may be controlled by an incompletely dominant gene. The inheritance pattern was consistent with that of barley *HvSS1* [36] but differed from the recessive inheritance of *OsEui* [37]. The further mapping and cloning of this mutant gene will enable the precise regulation of wheat plants’ height, thus aiding in enhancing the plant architecture and increasing the yield.

### 3.2. eui Causes the Elongation of the Peduncle Through Cell Elongation

The length of the peduncle is primarily determined by internode meristem division and cell elongation. The former primarily leads to changes in the number of cells, while the latter causes changes in the cell size. Previous studies have suggested that alterations in the cell size and number may result in changes in the plant height [38,39,40]. In this study, we found that *eui* promoted peduncle elongation mainly through internode cell elongation rather than through an increased number of cells, which is consistent with the findings of Wang [40].

### 3.3. The Elongation of eui Plants’ Peduncle May Be Mainly Regulated by Auxin

Integrating transcriptome and metabolome data facilitates a systematic analysis of biological systems, examining states and functions from the perspectives of gene expression and metabolite accumulation. Some studies have utilized multi-omics strategies to explore the mechanisms behind early stem thickness phenotypes in wheat, identifying the effects of the growth stage and allele on the expression of metabolites and transcripts related to stem solidity, cell wall development, and programmed cell death [41]. A multi-omics analysis of lignin biosynthesis and accumulation in wheat has offered insights into the variations in lodging resistance among different hybrid wheat varieties [13]. Our study also revealed the potential mechanisms involving auxin in peduncle elongation using multi-omics approaches. In the mutant, the auxin-related genes *DDC/TDC* and *ASMT*, which are part of the tryptophan biosynthesis pathway, were significantly upregulated. Tryptophan is a precursor for auxin synthesis and can be produced via various pathways, including the tryptamine, indole pyruvate, and indole acetonitrile pathways [42]. *DDC/TDC* is also involved in the tryptamine pathway. Through its action, tryptophan is converted into tryptamine, which subsequently transforms into IAA via the tryptamine pathway [43,44]. Both *TDC* and *ASMT* play roles in the biosynthesis of melatonin, a plant hormone that is crucial for controlling growth and various physiological responses [45]. The increased expression of these genes may lead to the accumulation of auxin metabolites. Our metabolome analysis indicated a significant elevation in auxin metabolite levels, including 4-CPA and IBA. Studies have suggested that the *TaPRR9* mutant exhibits phenotypes similar to those of the *eui* mutant, where the specific expression of photosynthetic genes and auxin-related genes in the peduncle promotes significant peduncle elongation during the heading stage [46]. *br2* mutants display a classic brachytic phenotype in maize, characterized by shortened stalk internodes without a reduction in the size of other plant parts, due to defects in the transport of the plant hormone auxin [47]. In this study, the elongation of the mutant peduncle may also have been related to changes in auxin levels. However, how auxin specifically regulates the elongation of the peduncle remains to be further investigated.

Numerous reports have indicated that gibberellin significantly enhances plant stem elongation [48,49,50]. However, although we observed upregulation in the expression of several gibberellin synthesis-related genes, such as *CPS* and *KS*, the majority of the GA_20OX_ and GA_3OX_ genes were downregulated, significantly decreasing the levels of active GA_7_ and GA_3_ in the mutant, with only a slight increase in GA_4_ at the S1 stage. It has been suggested that GA_4_ specifically regulates the elongation of the rice uppermost internode [51,52]. In this study, the *eui* mutant exhibited significant peduncle elongation, while other internodes showed little difference, which may have been related to this phenomenon. Brassinosteroids have also been reported to participate in the regulation of plant stem elongation [53,54]. In Arabidopsis, loss-of-function mutants of *DET2* exhibit growth inhibition phenotypes such as dwarfism [55]. The BR signaling-related genes *BZR* and *BIN2* also regulate stem elongation [56]. In this study, the upregulated expression of *DET2*, *BRI1*, and *BZR1_2* indicates their positive regulatory roles in peduncle elongation, while the downregulated expression of the *BIN2* gene further confirms its negative regulatory effect. Other plant hormones also regulate plant stem elongation [57,58], and we detected the differential expression of various other hormones. For instance, several CYT DEGs and DAMs were upregulated in the *eui* mutant, whereas genes related to the ABA levels were downregulated. Notably, genes and metabolites associated with JAs and SA in the mutant were also upregulated. Studies have reported that JAs and SA are the main plant hormones involved in regulating disease resistance [59]. This suggests that the presence of the mutant might enhance disease resistance due to peduncle elongation; however, further experimentation is required to confirm this.

In conclusion, the elongation of the peduncle in this mutant appears to be mostly regulated by auxin, which may interact with other hormones during the early heading stage. Considering that EMS mutagenesis can generate high-density point mutations, the DEGs and DAMs identified in this study may have been influenced by mutations at other loci. Further population development, including the creation and utilization of backcross populations, will assist in minimizing the background differences and aid in the mapping and cloning of mutant genes that are directly related to peduncle elongation. Nonetheless, multi-omics analysis can still provide valuable references for the mining of mutant genes and mechanistic analysis. Selected genes can also be used to develop functional markers and conduct association studies to determine their role in peduncle elongation, which can then be applied to molecular marker-assisted breeding.

## 4. Materials and Methods

### 4.1. Planting of Plant Materials and Measurement of Agronomic Traits

Originating from JM90 EMS mutagenesis, the stable *eui* mutant was obtained through successive generations of selfing and purification. From 2020 to 2024, the *eui* mutant, the wild-type (WT), and AK58 were planted in the experimental field at the College of Agriculture, Shanxi Agricultural University (37°25′ N, 112°25′ E). The row spacing was maintained at 0.25 m, while the plant spacing was set at 0.1 m. During the flowering stage, an AK58/*eui* hybrid combination was created, and F_1_ seeds were collected. Subsequently, the F_1_ generation was self-pollinated to produce the F_2_ generation. The plant height and peduncle length of each F_2_ individual were measured at maturity, and the segregation ratio was analyzed. At the maturity stage, various agronomic traits of *eui* plants and the WT were measured. These traits included the plant height, panicle length, peduncle length, length of other internodes, effective tiller number, spikelets per spike, and grain number per spike. After harvesting, the biological yield per plant, economic yield per plant, 1000-grain weight, grain length, grain width, and harvest index were calculated based on these measurements. We performed three biological replicates, each consisting of 10 plants with uniform growth.

### 4.2. Cell Tissue Sections and Microscopic Observation

At the S1 stage (prior to full spike emergence), there was no significant difference in peduncle elongation between *eui* plants and the WT. However, at the S2 stage (ten days post-S1, when spikes fully emerged), the peduncle of *eui* plants was significantly longer than that of the WT. At these stages, the peduncle elongated the most rapidly. The division of mesophytic tissue cells and the changes in the cell volume were also more pronounced. Therefore, we selected peduncles from these two stages for the microscopic observation of cellular structures. The middle portions of the peduncles from both *eui* plants and the WT at the S1 and S2 stages were cut into 1 cm segments and preserved in an FAA fixative. Following a sequence of dehydration, waxing, embedding, dewaxing, rehydration, and staining, the sections were fully examined using an optical microscope (Olympus, Tokyo, Japan) [39]. Subsequently, the internode cells were observed using Case Viewer 2.3. We performed three biological replicates. For each sample, we measured the lengths of 10 cells and took the average to determine the cell length.

### 4.3. Sampling for RNA Sequencing and Targeted Metabolome Analysis

Peduncle elongation coincides with the heading and flowering stages of wheat, further influencing its grain-filling capacity. To understand the gene expression levels during peduncle elongation, transcriptome sequencing was conducted on both *eui* and WT plants at the same developmental stage. Given the critical regulatory role of plant hormones in wheat stems’ elongation, targeted hormone metabolomics was performed concurrently. The peduncles of the *eui* and WT plants were collected as samples at the S1 and S2 stages and immediately stored in liquid nitrogen. For each sample, three replicates were prepared and labeled as follows: MS1 (S1 stage mutant), divided into M1, M2, and M3; WS1 (S1 stage WT), divided into W1, W2, and W3; MS2 (S2 stage mutant), divided into M4, M5, and M6; and WS2 (S2 stage WT), divided into W4, W5, and W6. In total, 12 samples were used for RNA extraction and hormone-targeted metabolite analysis.

### 4.4. RNA-seq Analysis

The total RNA was extracted from the tissue using TRIzol^®^ Reagent (ThermoFisher, Shanghai, China) according to the manufacturer’s instructions. The quality of the RNA was determined using a 5300 Bioanalyser (Agilent, Beijing, China) and quantified using the ND-2000 (NanoDrop, Wilmington, MA, USA). Only high-quality RNA samples were used to construct the sequencing library. RNA purification, reverse transcription, library construction, and sequencing were performed by Shanghai Majorbio Bio-pharm Biotechnology Co., Ltd. (Shanghai, China). The raw sequencing reads were initially cleaned by removing the adapter sequences and low-quality reads. The cleaned reads were then aligned with a cDNA database for the Chinese Spring wheat variety (IWGSC_RefSeq_v2.1). The gene and transcript expression levels were quantitatively analyzed using RSEM software Viewer 1.3.3, with the expression levels reported in FPKM (fragments per kilobase of the transcript per million mapped reads). DESeq2 software Viewer 1.24.0 was utilized to identify DEGs with a *p*-adjust value less than 0.05 and a fold change greater than 2 [60]. The KEGG (http://www.genome.jp/kegg, accessed on 12 December 2024) was used for the annotation and pathway enrichment analysis of the DEGs.

### 4.5. Targeted Metabolome Analysis

A total of 41 phytohormone standards were weighed accurately and dissolved in methanol. The solution was diluted to prepare mixed standard solutions with different concentrations and added to a 1.5 mL EP tube. A 100 mg sample was accurately weighed and added to a 2 mL centrifuge tube and mixed with 498 μL of 80% methanol and 2 μL of an internal standard solution (SA-D4, 2 μg/mL). Before extraction, a 6 mm grinding bead was added. Samples were then homogenated at −20 °C using a high-throughput tissue crusher (Wonbio-96c, Shanghai, China) operating at a frequency of 50 Hz for 3 min, followed by sonication at 40 kHz for 60 min at 5 °C. The samples were extracted using an EN15662 pack and shaken vigorously for 10 min. After centrifugation at 13,000× *g* at 10 °C for 10 min, the supernatant was injected into the LC-MS/MS system for analysis.

The LC-MS/MS analysis was conducted using an ExionLC AD system coupled with a QTRAP^®^ 6500+ mass spectrometer (Sciex, Redwood City, CA, USA) at Majorbio Bio-Pharm Technology Co., Ltd. (Shanghai, China) [61]. Briefly, the samples were separated using a Waters BEH C18 (2.1 × 100 mm, 1.7 μm), and a column oven was set to 30 °C. The separation of the metabolites was achieved at a 0.35 mL/min flow rate, with the mobile phase being a gradient consisting of 0.1% formic acid in water (solvent A) and 0.1% formic acid in acetonitrile (solvent B); the total chromatographic separation took 12 min.

The mass spectrometric data were collected using a UHPLC coupled with a QTRAP^®^ 6500+ mass spectrometer (Sciex, Redwood City, CA, USA) equipped with an electrospray ionization (ESI) source operating in a positive mode and negative mode. The source and gas settings were as follows: the source temperature was 550 °C; the curtain gas (CUR) was at 35 psi; the CAD gas pressure was medium; both Ion Source Gas 1 and Gas 2 were at 50 psi; and the ion spray voltage floating (ISVF) was −4500 V in the negative mode and 5500 V in the positive mode.

Quality control (QC) samples are mixed samples or concentrated mixed standard solutions used to assess the stability of an analytical system. QC samples were injected at regular intervals (every 10 samples) to examine the stability of the analysis; the RSD of the QC samples for the targets should have been less than 15%. The raw LC-MS data were imported into Sciex OS. All the ion fragments were automatically identified and integrated by using the default parameters, and all the integrations were checked manually. The metabolite concentration of each sample was calculated according to a linear regression standard curve. The concentration data matrix was uploaded to the Majorbio Cloud Platform (cloud.majorbio.com) for analysis [62]. Orthogonal partial least squares discriminant analysis (OPLS-DA) was performed using the R package ropls (version 1.6.2). The model stability was evaluated through seven-fold cross-validation. Statistically significant metabolites were identified using Student’s *t*-test (*p* < 0.05) and corrected for multiple comparisons. DAMs were identified based on the following criteria: a *p*-value of less than 1, a VIP_pre_OPLS-DA greater than 0, and a fold change greater than 1. Differentially accumulated metabolites were annotated against the KEGG pathway database (https://www.kegg.jp/kegg/pathway.html, accessed on 7 December 2024) to determine their associated metabolic pathways. Pathway enrichment analysis was conducted using the Python package scipy.stats, and the most relevant biological pathways linked to experimental treatments were identified using Fisher’s exact test.

### 4.6. Gene Expression Verification

Peduncles from both the *eui* plants and the WT at the S1 and S2 stages were collected, and the total RNA was extracted using the RNAiso Plus extraction reagent (TaKaRa, Dalian, China). For cDNA synthesis, we utilized the Fast Quant RT Kit with gDNase (Tiangen, Beijing, China). Each sample consisted of three biological replicates. Specific primers were designed using Primer5 and are shown in Supplementary Table S5. A quantitative RT-PCR (RT-qPCR) was conducted using the CFX96 fluorescence quantitative PCR machine (BIO-RAD, Hercules, CA, USA). The reaction mixture (10 μL) was composed of 1 μL of cDNA, 0.4 μL of the forward and reverse primers (10 μM each), 5 μL of TB Green Premix Ex Taq II, 0.2 μL of ROX, and 3 μL of dd H_2_O. Quantitative data were normalized using actin as the internal reference gene, and the relative expression levels were calculated using the formula 2^−ΔΔCt^ [63].

## 5. Conclusions

We compared the *eui* mutant and the wild-type (WT) in terms of their phenotypic characteristics, cytological observations, and the multi-omics analysis results. We found significant differences between them, including in the length of the peduncle, the number of spikelets per spike, and the number of grains per spike. This indicates that an elongated peduncle may affect the yield. Cytological observations revealed that peduncle elongation in the mutant was caused by cell elongation. Transcriptome and metabolomics analyses identified a series of *DDC/TDC* and *ASMT* auxin-related gene upregulations and 4-CPA, IAATrp, and IBA metabolite accumulation that may have promoted peduncle cell elongation. Except for GA_4_, the accumulation of other gibberellins was significantly lower than that in the WT, suggesting that the cell elongation in the mutant is primarily influenced by auxin. Overall, this study utilized a multi-omics approach to investigate the key metabolic pathways involved in common wheat peduncle elongation and to examine the effects of hormones on this process. The results offer a theoretical foundation for analyzing the molecular mechanisms behind wheat peduncle elongation, as well as for developing the ideal plant type and breeding high-yield varieties.

## Figures and Tables

**Table 1:** Inheritance of *eui* in wheat crosses with AK58.

Cross	Phenotype of F_2_	Observed Count (O)	Expected Count (E)	(O-E)2/E	χ2	Probable Ratio
AK58/*eui*	Mutation type	476	508	2.01575	2.704	1
	Intermediate type	1035	1016	0.35531		2
	Wild-type	521	508	0.33268		1
	Sum	2032	2032	2.70374		

## Data Availability

The data presented in this study are openly available from the NCBI. (https://www.ncbi.nlm.nih.gov/bioproject/, reference number PRJNA1241885, accessed on 26 March 2025).

## Supplementary Materials

The following supporting information can be downloaded at https://www.mdpi.com/article/10.3390/plants14111611/s1. Figure S1: Comparison of other agronomic traits between mutant *eui* and WT; Figure S2: Validation of the differentially expressed genes in the transcriptome; Figure S3: The DEGs and DAMs for the other hormones. Table S1: Comparison of sequencing data with reference genomes; Table S2: Main enrichment pathways from four different comparisons, referring to KEGG database; Table S3: Data table for 41 measured hormone metabolites; Table S4: Differential expression of auxin-related genes; Table S5: Differential expression of gibberellin-related genes; Table S6: Differential expression of other hormone-related genes; Table S7: Primers for quantitative real-time polymerase chain reaction.
