# Supplementary material for: Combining Transcriptome and Hormone-Targeted Metabolome Analyses to Dissect the Regulatory Mechanisms Underlying Wheat Peduncle Elongation
Source: Plants (Basel). 2025 May 25;14(11):1611. doi: 10.3390/plants14111611 (PMC12158073; doi:10.3390/plants14111611)
Supplement: Supplementary file 1 [file plants-14-01611-s001.zip › Figures S1-S3.pdf]

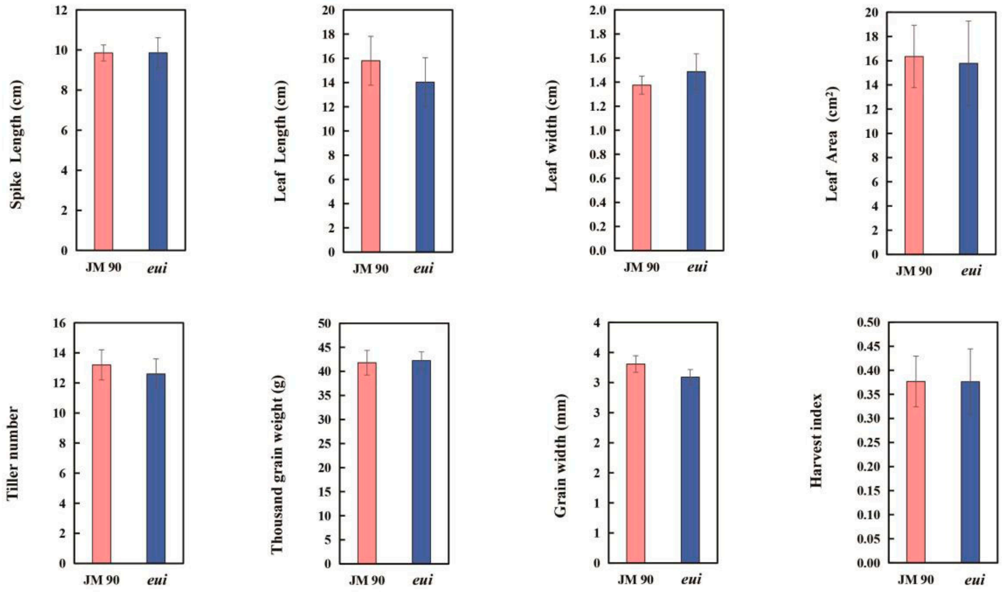

Figure S1. Comparison of other agronomic traits between the *eui* mutant and WT. The data represent the average of 10 plants, with an * indicating a significant difference between the wild-type JM90 and the mutant ($p < 0.05$, t-test).

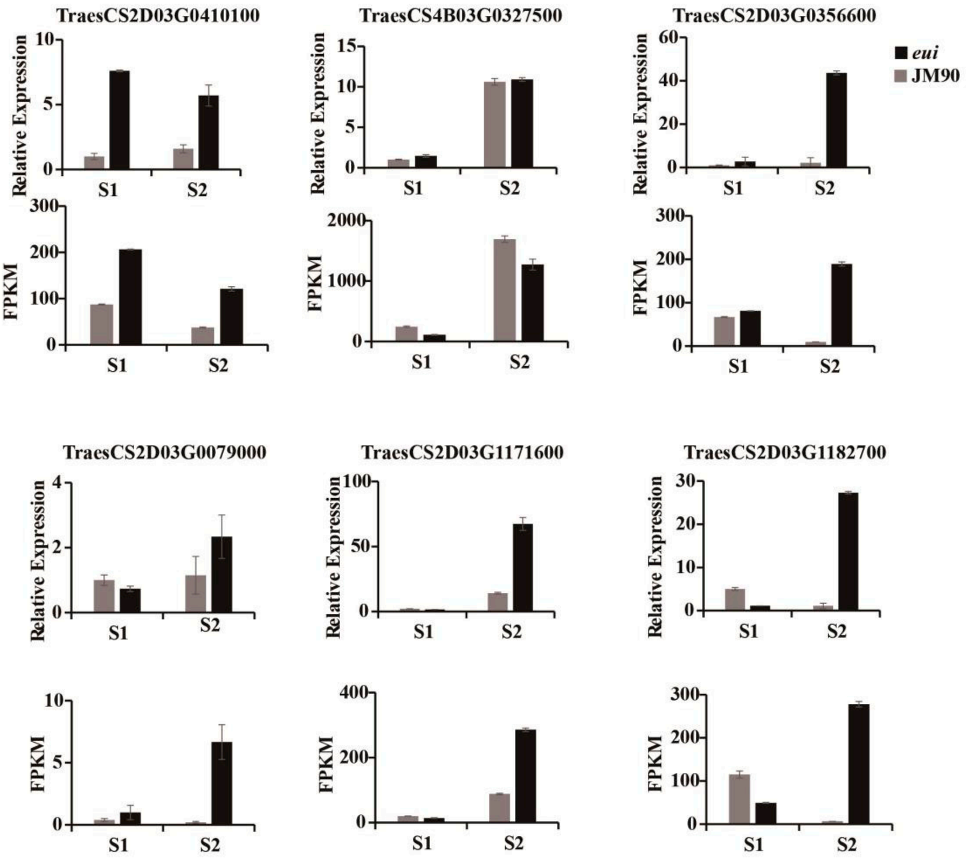

Figure S2. Validation of differentially expressed genes in transcriptome. The vertical coordinate labeled FPKM represents the transcriptome sequencing results, while the vertical coordinate labeled Relative Expression indicates the qRT-PCR results. The horizontal coordinate is the two measured stages, S1 and S2.

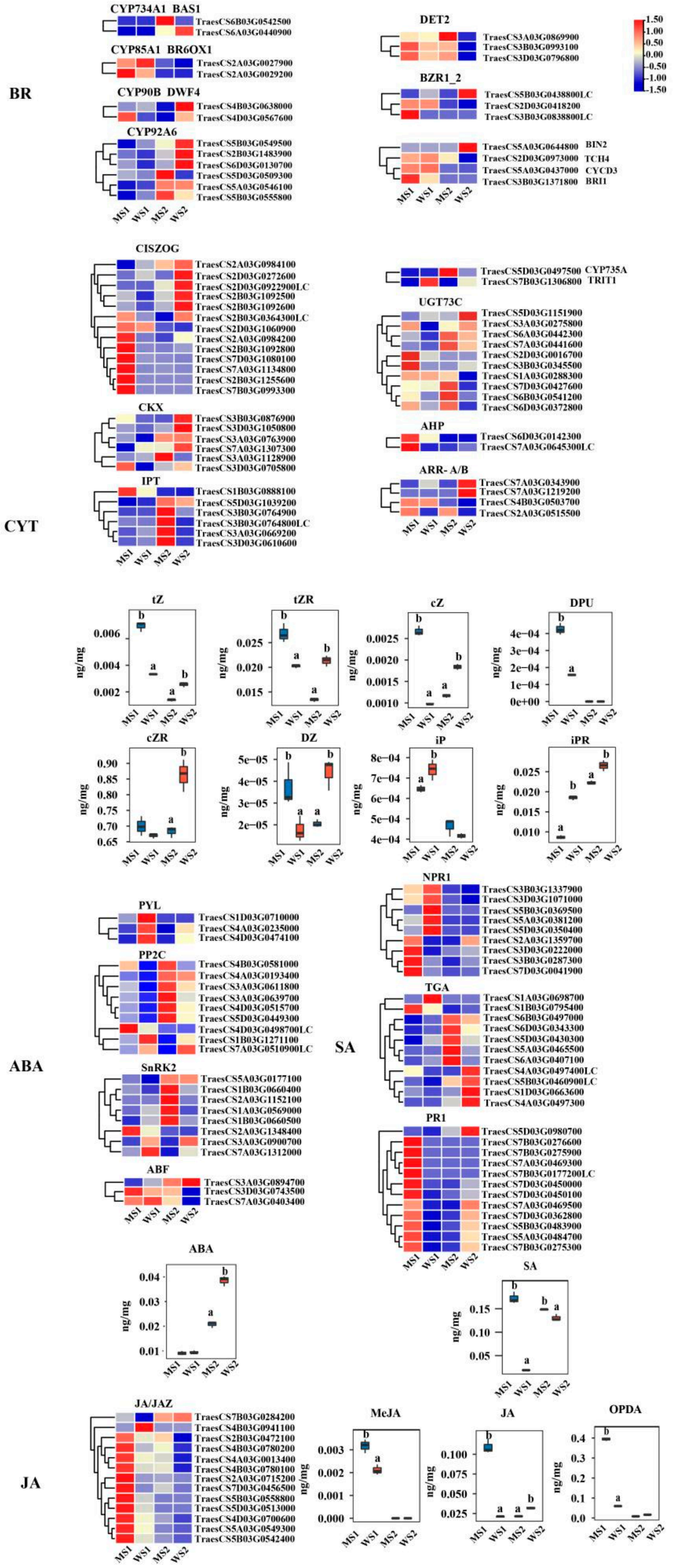
BR
CYP734A1 BAS1
TraesCS6B03G0542500
TraesCS6A03G0440900
CYP85A1 BR6OX1
TraesCS2A03G0027900
TraesCS2A03G0029200
CYP90B DWF4
TraesCS4B03G0638000
TraesCS4D03G0567600
CYP92A6
TraesCS5B03G0549500
TraesCS2B03G1483900
TraesCS6D03G0130700
TraesCS5D03G0509300
TraesCS5A03G0546100
TraesCS5B03G0555800
DET2
TraesCS3A03G0869900
TraesCS3B03G0993100
TraesCS3D03G0796800
BZR1_2
TraesCS5B03G0438800LC
TraesCS2D03G0418200
TraesCS3B03G0838800LC
TraesCS5A03G0644800 BIN2
TraesCS2D03G0973000 TCH4
TraesCS5A03G0437000 CYCD3
TraesCS3B03G1371800 BRI1
1.50
1.00
0.50
0.00
-0.50
-1.00
-1.50
CYT
CISZOG
TraesCS2A03G0984100
TraesCS2D03G0272600
TraesCS2D03G0922900LC
TraesCS2B03G1092500
TraesCS2B03G1092600
TraesCS2B03G0364300LC
TraesCS2D03G1060900
TraesCS2A03G0984200
TraesCS2B03G1092800
TraesCS7D03G1080100
TraesCS7A03G1134800
TraesCS2B03G1255600
TraesCS7B03G0993300
CKX
TraesCS3B03G0876900
TraesCS3D03G1050800
TraesCS3A03G0763900
TraesCS7A03G1307300
TraesCS3A03G1128900
TraesCS3D03G0705800
IPT
TraesCS1B03G0888100
TraesCS5D03G1039200
TraesCS3B03G0764900
TraesCS3B03G0764800LC
TraesCS3A03G0669200
TraesCS3D03G0610600
TraesCS5D03G0497500 CYP735A
TraesCS7B03G1306800 TRIT1
UGT73C
TraesCS5D03G1151900
TraesCS3A03G0275800
TraesCS6A03G0442300
TraesCS7A03G0441600
TraesCS2D03G0016700
TraesCS3B03G0345500
TraesCS1A03G0288300
TraesCS7D03G0427600
TraesCS6B03G0541200
TraesCS6D03G0372800
AHP
TraesCS6D03G0142300
TraesCS7A03G0645300LC
ARR- A/B
TraesCS7A03G0343900
TraesCS7A03G1219200
TraesCS4B03G0503700
TraesCS2A03G0515500
MS1 WS1 MS2 WS2
tZ
tZR
cZ
DPU
cZR
DZ
iP
iPR
ng/mg
ABA
PYL
TraesCS1D03G0710000
TraesCS4A03G0235000
TraesCS4D03G0474100
PP2C
TraesCS4B03G0581000
TraesCS4A03G0193400
TraesCS3A03G0611800
TraesCS3A03G0639700
TraesCS4D03G0515700
TraesCS5D03G0449300
TraesCS4D03G0498700LC
TraesCS1B03G1271100
TraesCS7A03G0510900LC
SnRK2
TraesCS5A03G0177100
TraesCS1B03G0660400
TraesCS2A03G1152100
TraesCS1A03G0569000
TraesCS1B03G0660500
TraesCS2A03G1348400
TraesCS3A03G0900700
TraesCS7A03G1312000
ABF
TraesCS3A03G0894700
TraesCS3D03G0743500
TraesCS7A03G0403400
SA
NPR1
TraesCS3B03G1337900
TraesCS3D03G1071000
TraesCS5B03G0369500
TraesCS5A03G0381200
TraesCS5D03G0350400
TraesCS2A03G1359700
TraesCS3D03G0222000
TraesCS3B03G0287300
TraesCS7D03G0041900
TGA
TraesCS1A03G0698700
TraesCS1B03G0795400
TraesCS6B03G0497000
TraesCS6D03G0343300
TraesCS5D03G0430300
TraesCS5A03G0465500
TraesCS6A03G0407100
TraesCS4A03G0497400LC
TraesCS5B03G0460900LC
TraesCS1D03G0663600
TraesCS4A03G0497300
PR1
TraesCS5D03G0980700
TraesCS7B03G0276600
TraesCS7B03G0275900
TraesCS7A03G0469300
TraesCS7B03G0177200LC
TraesCS7D03G0450000
TraesCS7D03G0450100
TraesCS7A03G0469500
TraesCS7D03G0362800
TraesCS5B03G0483900
TraesCS5A03G0484700
TraesCS7B03G0275300
JA
JA/JAZ
TraesCS7B03G0284200
TraesCS4B03G0941100
TraesCS2B03G0472100
TraesCS4B03G0780200
TraesCS4A03G0013400
TraesCS4B03G0780100
TraesCS2A03G0715200
TraesCS7D03G0456500
TraesCS5B03G0558800
TraesCS5D03G0513000
TraesCS4D03G0700600
TraesCS5A03G0549300
TraesCS5B03G0542400
MeJA
OPDA

Figure S3. The DEGs and DAMs of other hormones. Note: The relative level of expression is shown by a color gradient from low (blue) to high (red). The blocks are arranged from left to right: MS1, WS1, MS2, WS2. Numbers in the scale represents the FPKM values of gene expression after standardization.The metabolite boxplot displays the content distribution of metabolites in each sample group. The line in the middle of the box represents the median of the relative expression abundance of the substance. Statistically significant metabolites were identified using Student's t-test ($p < 0.05$) and corrected for multiple comparisons.Different letters represent a significant difference between the mutant and the wild-type in the same stage. BR: brassinosteroid; CYT: cytokinins; ABA: abscisic acid; SA: sallcylic acid; JA: jasmonicacid; *CYP734A1/BAS1:brahma-associated SWI/SNF complexes; CYP85A1/BR6OX1:brassinosteroid-6-oxidases1; CYP90B/DWF4: Dwarf4; CYP92A6:cytochrome P450 92 A6; DET2: Deetiolated 2; BZR1_2: brassinazole resistant 1_2; BIN2: brassinosteroid insensitive 2; TCH4: xyloglucan endotransglycosylase 4; CYCD3: D-type cyclin family 3; BRI1: BR receptor kinase; CISZOG: cis-zeatin-O-glucosyltransferase; CKX: cytokinin oxidase/dehydrogenase; IPT: isopentenyl transferases; CYP735A: cytochrome P450 735 A-like; TRIT1: TRNA isopentenyltransferase 1; UGT73C: UDP glycosyltransferase; AHP: arabidopsis histidine-phosphotransfer proteins; ARR-A/B: arabidopsis response regulator A/B; ABF: abscisic acid-responsive transcription factors; PP2C: protein phosphatase 2C; PYL: abscisic acid receptor PYL4-like; SnRK2: SNF1-related protein kinase 1; JAZ: jasmonate-zim-domain protein; NPR1: nonexpressor of pathogenesis-related genes 1; PR1:* pathogenesis-related protein; *TGA: TGACG-binding factor*; tZ: trans-Zeatin; tZR: trans-zeatin-riboside; cZ: cis-zeatin; cZR: cis-zeatin-riboside; DZ: dihydrozeatin; DPU: N,N'-diphenylurea; iP: N6-isopentenyladenine; iPR: N6-(Δ2-isopentenyl) adenosine; MeJA: methyl jasmonate; OPDA: 12-oxophytodienoic acid.
